# Association of Exposure to Court-Ordered Tobacco Industry Antismoking Advertisements With Intentions and Attempts to Quit Smoking Among US Adults

**DOI:** 10.1001/jamanetworkopen.2020.9504

**Published:** 2020-07-07

**Authors:** Onyema Greg Chido-Amajuoyi, Israel Agaku, Dale S. Mantey, Robert K. Yu, Sanjay Shete

**Affiliations:** 1 Department of Epidemiology, The University of Texas MD Anderson Cancer Center, Houston; 2 Department of Oral Health Policy and Epidemiology, Harvard School of Dental Medicine, Boston, Massachusetts; 3 Department of Health Promotion and Behavioral Science, University of Texas School of Public Health, Houston; 4 Department of Biostatistics, The University of Texas MD Anderson Cancer Center, Houston; 5 Division of Cancer Prevention and Population Sciences, The University of Texas MD Anderson Cancer Center, Houston

## Abstract

**Importance:**

In 2006, a US district court judge ordered tobacco companies to sponsor nationwide antismoking advertising campaigns. This landmark ruling and its subsequent execution represent an unprecedented tobacco control event; however, the association of this campaign with intentions and/or attempts to quit smoking is unknown.

**Objectives:**

To assess the reach of the expanded court-ordered tobacco industry antismoking advertisements (via television, newspapers, tobacco company websites, and/or cigarette packages), to examine associations between exposure to industry antismoking advertisements and intentions and/or attempts to quit smoking among cigarette smokers, and to calculate the numbers of US smokers who would have quit intentions associated with exposure to multiple advertisements.

**Design, Setting, and Participants:**

Data for this study were obtained from 5309 US adults, including 610 smokers, who responded to the Health Information National Trends Survey, a nationally representative cross-sectional survey conducted from January 22 to April 30, 2019. Respondents were representatives of households selected by equal-probability sampling of a database of US residential addresses.

**Exposure:**

Reported exposure to antismoking messages.

**Main Outcomes and Measures:**

Cigarette smoking cessation attempt in the past 12 months and intentions to quit cigarette smoking in the next 6 months. Covariates were age, sex, household annual income, race/ethnicity, educational level, and geographical residence. Data were weighted to be nationally representative after applying survey weights specified for the survey cycle.

**Results:**

The overall sample of 5309 respondents were a mean (SD) age of 55.6 (19.1) years and included 3073 women (51.2%), 3037 non-Hispanic white respondents (59.1%), 4645 respondents who lived in urban US areas (84.7%), and 610 current smokers (12.5%). Findings indicate that 2464 US adults (45.8%; 95% CI, 43.2%-48.5%) and 410 current smokers (66.8%; 95% CI, 61.1%-72.4%) were exposed to antismoking advertisements. Exposure to multiple antismoking messages was associated with 2.19 (95% CI, 1.10-4.34) greater odds of having intentions to quit cigarette smoking but was not associated with attempts to quit (adjusted odds ratio, 1.31; 95% CI, 0.69-2.52). Furthermore, an examination of the association of cumulative exposure to antismoking messages with cessation intentions revealed that, with each additional exposure to an antismoking message, the odds of smoking cessation intentions increased by 1.21 (95% CI, 1.02-1.44). If all smokers were to be exposed to multiple antitobacco messages, there could be an estimated 3.98 million (95% CI, 492 480-7 223 040) current smokers in the United States with intentions to quit.

**Conclusions and Relevance:**

Although the reach of court-ordered industry advertisements increased among smokers, the reach of these advertisements within the general population remains suboptimal. The finding that industry advertisements helped smokers consider quitting highlights their potential to aid smoking cessation. However, the lack of association with actual attempts to quit suggests that the industry antismoking advertisement campaigns were inadequate. The design and content of industry antismoking advertisement campaigns should be enhanced to help smokers quit.

## Key Points

**Question:**

Is exposure to tobacco industry antismoking advertisements associated with increased odds of smoking cessation intentions and/or attempts among current smokers?

**Findings:**

In a cross-sectional survey of US adults, less than half reported seeing tobacco industry antismoking advertisements via television, newspapers, tobacco company websites, or cigarette packages. Exposure to antismoking messages was associated with increased odds of intentions to quit smoking among smokers, but no associations were found with actual attempts to quit smoking.

**Meaning:**

Industry-sponsored antismoking advertisements were associated with increased intentions to quit smoking; however, they have not been effective in stimulating attempts to quit among current smokers.

## Teaser

This survey study assesses the reach of court-ordered tobacco industry antismoking advertisements the association between exposure to these advertisements and intentions and/or attempts to quit smoking among cigarette smokers.

## Introduction

In 1999, the US Department of Justice filed a lawsuit against the tobacco industry for misleading the general public on the dangers of tobacco use, thereby violating the Racketeer Influenced and Corrupt Organizations Act.^1^ In proceedings that followed this suit, US District Judge Gladys Kessler ordered the tobacco industry to make “corrective statements” about the hazards of tobacco use.^1^ To this end, corrective advertising campaigns were executed via newspapers (from November 2017 to May 2018) and television (from November 2017 to November 2018); court-ordered corrective messaging placement on tobacco company corporate websites (beginning June 2018) and as onserts on cigarette packages (beginning November 2018) are currently ongoing.^2,3^ Antismoking advertisement placement at retail points of sale are currently the subject of ongoing litigation.^4^

Antismoking mass media campaigns are a key component of tobacco control strategies recommended by the World Health Organization.^5,6,7^ Exposure to antismoking advertising has been shown to be associated with decreased smoking prevalence, as well as increased likelihood of smoking cessation intentions and attempts.^8,9,10,11,12,13^ Studies suggest that media channel type^11^; source (government, pharmaceutical, and tobacco industry)^9,14^; content, tone, and intensity^15,16,17,18^; and dose, frequency, and duration^17,19^ of antismoking campaigns are significantly associated with smoking-related outcomes. Furthermore, cumulative exposure to antismoking messages via multiple channels increases the likelihood of a subsequent attempt to quit smoking.^10^

The original corrective antismoking messages were preambled by the statement, “A federal court has ruled that R. J. Reynolds Tobacco, Philip Morris USA, Altria and Lorillard deliberately deceived the American public about the health effects of smoking and has ordered those companies to make this statement. Here is the truth: [topic/theme of corrective statement],”^2,20^^(p570)^ which conveyed the industry’s guilt. However, the tobacco industry successfully appealed to have key components of this message modified. These modifications may have weakened the corrective potency of the final version of the corrective statements.^2,20^

A study of the coverage of the television and newspaper portions of the advertising campaign revealed that only 40% of the US adult population saw the antismoking advertisements and that coverage was even lower among groups known to be at risk of tobacco use, such as youths and socioethnic minority groups.^21^ However, since the completion of the newspaper and television components of the court-ordered advertising campaign and the onset of the implementation of a corrective statement placement on tobacco company websites and cigarette packages, no assessment has been conducted on the population-level reach of the overall antismoking advertising campaigns, to our knowledge. In addition, previous data did not allow for individual-level assessment of the outcome of this advertising campaign, such as its association with smoking cessation intentions or attempts.

This study aims to examine the reach of the fully completed television and newspaper antismoking advertisements, as well as the ongoing placement of antismoking messages on tobacco company websites and cigarette packages. Furthermore, we examine the association between exposure to corrective antismoking messages and predecessors of smoking cessation among a representative sample of adult cigarette smokers in the United States. Specifically, this study examines the association between cumulative exposure to corrective antismoking messages and intentions to quit cigarette smoking in the next 6 months. In addition, this study examines the association between cumulative exposure to corrective antismoking messages and attempts to quit in the past 12 months. We hypothesize that cumulative exposure to corrective antismoking messages will be significantly associated with greater odds of both past attempts to quit smoking and intentions to quit smoking in the next 6 months. Secondarily, we sought to estimate the potential real-world effect of being exposed to multiple antitobacco messages concurrently. This is critical because, while specific antismoking campaigns may only measure and evaluate the outcome of their own intervention, the reality is that the public is exposed to multiple advertisements from different sources.

## Methods

### Study Population and Design and Setting

Data for this study were obtained from the Health Information National Trends Survey (HINTS 5; cycle 3), a National Cancer Institute–administered survey of US adults that is nationally representative, conducted from January 22 to April 30, 2019. The sample frame for HINTS 5 cycle 3 was derived from a database of US residential addresses provided by Marketing Systems Group. In this sampling frame, addresses were grouped into low-minority and high-minority strata. The high-minority strata were oversampled to enhance accuracy of estimates for this population. The sampling strategy for HINTS 5 cycle 3 had 2 phases. First, an equal-probability method was used to select addresses within each stratum, and, second, an adult was selected per sampled household. Written informed consent was obtained from study participants. HINTS 5 cycle 3 was approved by the Westat Institutional Review Board and classified as exempt from review by the US National Institutes of Health Office of Human Subjects Research Protections because the data were deidentified. A detailed description of the survey method has been published.^22^ This study followed the reporting recommendations of the Strengthening the Reporting of Observational Studies in Epidemiology (STROBE) reporting guideline.^23^

A web survey was piloted, but respondents were also given the option to the complete survey via a paper questionnaire. Paper questionnaire responses were returned by mail and were documented by month and period of return. The response rate for cycle 3 (paper only) was 30.2% (n = 3439), while the web pilot study response rate was 30.6% (n = 2035). The overall response rate for HINTS 5 cycle 3 was 30.3% (n = 5474). This was estimated using the RR2 formula of the American Association of Public Opinion Research^24^ and is comparable to those of previous cycles of HINTS.^25^

### Study Variables

#### Exposure Measure

The main exposure measure of this study was self-reported exposure to court-ordered antismoking messages, as well as the scale of exposure to the different corrective messages. Exposure to corrective messages was via media that includes television, newspapers, tobacco company corporate websites, or cigarette package onserts. To assess exposure, respondents were first asked, “In the past 12 months, have you seen messages saying that a federal court has ordered tobacco companies to make statements about the dangers of smoking cigarettes? These messages have been in newspapers, on television, on tobacco company websites, and on cigarette packs?”

Respondents who answered “yes” were then assessed for the type of corrective message seen using the question: “Which of the following messages about the dangers of smoking cigarettes have you seen?” Possible responses were: “federal court–ordered tobacco messages: health effects of smoking,” “federal court–ordered tobacco messages: health effects secondhand smoke,” “federal court–ordered tobacco messages: addictiveness,” “federal court–ordered tobacco messages: enhanced delivery,” and “federal court–ordered tobacco message: low-tar and light cigarettes.”

Respondents who answered “no” to the initial question were categorized to “no message seen.” Respondents who indicated having seen only 1 of the 5 corrective messages were categorized to “single message seen.” Respondents who selected more than 1 type of corrective message were categorized to “multiple messages seen.”

#### Outcome Measure

We examined predecessors of smoking cessation among current smokers. We assessed smoking cessation intentions using the following survey question: “Are you seriously considering quitting smoking within the next 6 months?” Respondents who answered “yes” were considered to have intentions to quit. Smoking cessation attempts were assessed using the following questions: “At any time in the past year, have you stopped smoking for 1 day or longer because you were trying to quit?” Responses of “yes” were considered attempts to quit.

Outcome questions were restricted to current smokers. This population was derived from respondents who answered “yes” to the survey question “Have you smoked at least 100 cigarettes in your entire life?” and reported smoking “every day” or “some days” when asked: “Do you now smoke cigarettes every day, some days, or not at all?”

#### Covariates

The sociodemographic characteristics of study respondents were used as covariates, including age, sex, household annual income, race/ethnicity, educational level, and rural or urban residence. Age was grouped into 4 categories: 18 to 34 years, 35 to 49 years, 50 to 64 years, and 65 years or older. Race/ethnicity was categorized as non-Hispanic white, non-Hispanic black, and Hispanic. Educational level was grouped into 3 categories: high school graduate or lower, post–high school or some college (a combination of post–high school training other than college and some college training), and college graduate or postgraduate. Household annual income was categorized as less than $35 000, $35 000 to $49 999, $50 000 to $74 999, and $75 000 or more. Residence was defined using the US Department of Agriculture’s 2003 Rural-Urban Continuum Codes. Codes 1 to 3 were designated as urban, and codes 4 to 9 were categorized as rural.

### Statistical Analysis

Data were weighted to be nationally representative after applying survey weights specified for HINTS 5 cycle 3. Specifically, data were calibrated using the estimates from the 2017 American Community Survey of the US Census Bureau. The computation of full-sample weights consisted of calculating household-level base weights, adjusting for household nonresponse, calculating person-level initial weights, and calibrating the person-level weights to population counts. Further details on the weighting methods is described in the survey methodology report.^26^ The HINTS 5 cycle 3 data release provided final sample weights for the calculation of estimates as well as replicate weights for the calculation of the SE of estimates. We used the final weights in the *weight* option and replicated weights in the *repweight* option of the PROC SURVEYLOGISTIC Statement (SAS, version 9.4; SAS Institute Inc) as recommended by HINTS.

Prevalence and associated 95% CIs of exposure to the court-ordered antismoking messages via newspaper, television, tobacco company corporate websites, or cigarette package were estimated for the overall adult population by age, sex, household annual income, race/ethnicity, educational level, and rural or urban residence. We also conducted prevalence estimates of advertisement exposure among a subpopulation of current smokers.

Prevalence and associated 95% CIs of current smokers with smoking cessation intentions and attempts were also estimated by advertisement exposure status (no message seen, single message seen, or multiple messages seen), by mean number of corrective message types seen, and by the sociodemographic characteristics outlined. Survey-weighted logistic regressions were used to study the association between exposure to court-ordered industry antismoking messages and intentions and attempts to quit among US adult smokers. An additional survey-weighted logistic regression analysis was conducted to determine whether cumulative exposure to different types of antismoking messages (by summing the number of antismoking messages seen) increased the odds of smoking cessation intentions or attempts. Statistical significance was defined as *P* < .05, and all tests were 2-tailed. Analyses were also conducted after stratifying current smokers into those who smoke every day and those who smoke some days.

To model the potential real-world effect of adults being exposed to multiple messages, we calculated the adjusted odds ratios associated with being exposed to multiple corrective statements (assuming homogeneity in treatment effects between these messages and any other messages). To calculate the excess numbers of individuals who would have a quit intention assocated with exposure to multiple advertisements, we multiplied the estimated excess risk by the numbers of US adults who had a quit intention (number of US cigarette smokers, 34.2 million,^27^ multiplied by the probability of having a quit intention, estimated from the current data). All data were weighted to be nationally representative and analyzed with SAS, version 9.4.

## Results

The overall sample of 5309 respondents were a mean (SD) age of 55.6 (19.1) years and included 3073 women (51.2%), 3037 non-Hispanic white respondents (59.1%), 4645 respondents who lived in urban US areas (84.7%), and 610 current smokers (12.5%) (Table 1). A total of 2464 of the overall study sample (45.8%; 95% CI, 43.2%-48.5%) reported exposure to US federal court–ordered antismoking advertisements. Exposure was lowest among respondents who had a high school education or lower (534 [41.7%; 95% CI, 36.1%-47.3%]), respondents who had a household annual income less than $35 000 (661 [40.6%; 95% CI, 35.1%-46.0%]), respondents who were aged 35 to 49 years (417 [41.1%; 95% CI, 36.9%-45.4%]), and respondents who were Hispanic (302 [43.2%; 95% CI, 36.1%-50.3%]). Among the subpopulation of 610 current smokers (Table 2), 410 (66.8%; 95% CI, 61.1%-72.4%) reported seeing court-ordered antismoking messages. Exposure rates were lowest among Hispanic individuals (36 [43.2%; 95% CI, 23.3%-63.1%]).

**Table 1.** Exposure to US Federal Court–Ordered Antismoking Advertisements Among US Adults, by Sociodemographic Characteristics and Smoking Status

Characteristic	Overall sample, No.	Exposure to advertisements, No. (%; 95% CI)^a^
Total	5309	2464 (45.8; 43.2-48.5)
Sex		
Male	2236	1053 (47.4; 42.6-52.3)
Female	3073	1411 (44.3; 41.8-46.7)
Age, y		
18-34	670	311 (49.5; 41.5-57.6)
35-49	951	417 (41.1; 36.9-45.4)
50-64	1642	819 (48.3; 43.8-52.7)
≥65	1937	867 (44.4; 41.4-47.5)
Race/ethnicity		
Non-Hispanic white	3037	1457 (48.6; 45.6-51.6)
Non-Hispanic black	672	334 (43.9; 37.4-50.4)
Hispanic	722	302 (43.2; 36.1-50.3)
Educational level		
High school graduate or lower	1261	534 (41.7; 36.1-47.3)
Post–high school or some college	1574	753 (48.9; 45.3-52.6)
College graduate or postgraduate	2399	1148 (45.4; 41.7-49.2)
Residence		
Urban	4645	2163 (45.7; 42.7-48.7)
Rural	664	301 (46.5; 40.1-52.8)
Household annual income, $		
<35 000	1497	661 (40.6; 35.1-46.0)
35 000-49 999	625	298 (46.1; 38.2-53.9)
50 000-74 999	843	397 (44.3; 38.1-50.5)
≥75 000	1796	872 (50.2; 47.0-53.4)
Smoking status		
Current^b^	610	410 (66.8; 61.1-72.4)
Former	1397	686 (49.3; 45.3-53.2)
Never	3253	1349 (40.5; 36.8-44.1)

^a^ Indicates “yes” response to the survey question: “In the past 12 months, have you seen messages saying that a Federal Court has ordered tobacco companies to make statements about the dangers of smoking cigarettes? These messages have been in newspapers, on television, on tobacco company websites, and on cigarette packs?”

^b^ Seven current smokers had missing information on sex and were removed from the analyses.

**Table 2.** Exposure to US Federal Court–Ordered Antismoking Advertisements Among Current Smokers, by Sociodemographic Characteristics

Characteristic	Overall sample, No.	Exposure to advertisements, No. (%; 95% CI)^a^
Total	610	410 (66.8; 61.1-72.4)
Sex		
Male	283	193 (68.9; 59.3-78.5)
Female	327	217 (64.2; 57.6-70.8)
Age, y		
18-34	57	34 (69.2; 53.5-84.9)
35-49	124	78 (58.5; 47.0-69.9)
50-64	267	186 (71.7; 62.9-80.6)
≥65	154	107 (70.2; 58.7-81.8)
Race/ethnicity		
Non-Hispanic white	338	237 (71.9; 64.3-79.6)
Non-Hispanic black	90	66 (59.0; 42.5-75.4)
Hispanic	72	36 (43.2; 23.3-63.1)
Educational level		
High school graduate or lower	218	135 (62.0; 51.3-72.7)
Post–high school or some college	225	159 (72.7; 64.2-81.3)
College graduate or postgraduate	162	113 (61.6; 46.5-76.7)
Residence		
Urban	516	342 (68.2; 62.1-74.3)
Rural	94	68 (60.1; 43.1-77.2)
Household annual income, $		
<35 000	283	182 (57.4; 46.9-67.9)
35 000-49 999	88	65 (71.3; 58.9-83.7)
50 000-74 999	69	44 (67.5; 54.8-80.1)
≥75 000	119	84 (80.4; 69.1-91.7)

^a^ Indicates “yes” response to the survey question: “In the past 12 months, have you seen messages saying that a Federal Court has ordered tobacco companies to make statements about the dangers of smoking cigarettes? These messages have been in newspapers, on television, on tobacco company websites, and on cigarette packs?”

Among current smokers, 389 (60.0%; 95% CI, 53.7%-66.3%) were seriously considering quitting smoking within the next 6 months, while 368 (60.6%; 95% CI, 55.2%-65.9%) had made quit attempts at any time within the past year (Table 3). Smoking cessation intentions were reported among current smokers who were unexposed to antismoking messages (123 [53.0%; 95% CI, 40.5%-65.5%]) but were higher among those who were exposed to one (79 [55.6%; 95% CI, 40.4%-70.7%]) or multiple (172 [65.8%; 95% CI, 57.5%-74.0%]) antismoking messages. Similarly, smoking cessation attempts were reported among current smokers who were unexposed to antismoking messages (123 [56.7%; 95% CI, 44.1%-69.2%]) but were higher among those exposed to one (73 [62.7%; 95% CI, 49.7%-75.7%]) or multiple (158 [60.0%; 95% CI, 50.7%-69.4%]) antismoking messages. Descriptive results of smoking cessation intentions and attempts of current smokers, stratified by use of cigarettes every day or some days, are provided in eTable 1 and eTable 2 in the Supplement. The associations of advertisement exposure with the characteristics of respondents are reported in eTable 3 in the Supplement.

**Table 3.** Smoking Cessation Intentions and Attempts Among Current Smokers, by Sociodemographic Characteristics and Antismoking Advertisement Exposure

Characteristic	Smoking cessation intention		Smoking cessation attempted	
	Overall sample, No.	Yes, No. (%; 95% CI)^a^	Overall sample, No.	Yes, No. (%; 95% CI)^b^
Total	599	389 (60.0; 53.7-66.3)	606	368 (60.6; 55.2-65.9)
Sex				
Male	280	185 (60.0; 50.3-69.7)	282	174 (62.0; 53.1-70.9)
Female	319	204 (60.1; 51.2-69.0)	324	194 (58.9; 50.9-66.9)
Age, y				
18-34	57	29 (51.6; 32.9-70.2)	57	40 (74.9; 61.9-87.9)
35-49	122	88 (68.9; 57.3-80.6)	123	75 (60.9; 47.6-74.2)
50-64	265	175 (58.0; 48.4-67.7)	266	153 (57.9; 50.0-65.8)
≥65	150	94 (54.7; 41.9-67.5)	153	95 (53.8; 41.3-66.2)
Race/ethnicity				
Non-Hispanic white	336	203 (55.4; 47.1-63.6)	337	183 (54.4; 46.6-62.2)
Non-Hispanic black	89	64 (59.9; 42.0-77.8)	89	65 (68.3; 53.0-83.5)
Hispanic	71	44 (67.2; 52.2-82.1)	72	45 (70.9; 55.0-86.7)
Educational level				
High school graduate or lower	213	132 (58.8; 48.7-69.0)	217	118 (50.3; 41.4-59.2)
Post–high school or some college	221	140 (59.0; 48.5-69.5)	223	144 (65.1; 54.9-75.3)
College graduate or postgraduate	160	114 (62.6; 48.2-77.1)	161	102 (71.1; 58.6-83.7)
Residence				
Urban	506	336 (62.0; 55.8-68.3)	512	322 (64.7; 59.7-69.8)
Rural	93	53 (50.8; 33.1-68.6)	94	46 (41.7; 25.3-58.2)
Household annual income, $				
<35 000	281	189 (57.3; 44.9-69.6)	282	180 (61.3; 51.6-71.0)
35 000-49 999	86	57 (65.6; 48.8-82.4)	87	56 (68.0; 53.7-82.3)
50 000-74 999	68	42 (62.3; 44.8-79.9)	69	37 (52.0; 35.0-69.0)
≥75 000	117	79 (62.8; 50.7-74.8)	118	68 (61.3; 49.1-73.4)
Exposure to antismoking advertisements				
Not seen	195	123 (53.0; 40.5-65.5)	198	123 (56.7; 44.1-69.2)
Single message type seen	120	79 (55.6; 40.4-70.7)	121	73 (62.7; 49.7-75.7)
Multiple message types seen	263	172 (65.8; 57.5-74.0)	265	158 (60.0; 50.7-69.4)
Exposure to antismoking advertisements				
No. of messages seen	578	374 (1.80; 1.50-2.00)^c^	584	354 (1.60; 1.40-1.80)^c^

^a^ Indicates “yes” response to the survey question: “Are you seriously considering quitting smoking within the next 6 months?”

^b^ Indicates “yes” response to the survey question: “At any time in the past year, have you stopped smoking for 1 day or longer because you were trying to quit?”

^c^ Mean (95% CI) number of messages seen by participants.

In our multivariate logistic regression analysis (Table 4), after adjusting for covariates, we found that exposure to multiple antismoking messages was associated with increased odds of intentions to quit smoking (adjusted odds ratio, 2.19; 95% CI, 1.10-4.34); however, exposure to a single antismoking message was not associated with smoking cessation intention (adjusted odds ratio, 1.22; 95% CI, 0.50-3.01). Also, exposure to a single corrective message (adjusted odds ratio, 1.37; 95% CI, 0.64-2.92) or multiple corrective messages (adjusted odds ratio, 1.31; 95% CI, 0.69-2.52) was not associated with smoking cessation attempts. Finally, we found that, with each additional exposure to a corrective antismoking message, the odds of smoking cessation intentions among cigarette smokers increased by 1.21 (95% CI, 1.02-1.44), adjusting for covariates. Once again, additive exposure to multiple corrective messages was not associated with smoking cessation attempts (adjusted odds ratio, 1.00; 95% CI, 0.84-1.20). Results of analyses after stratifying current smokers by use of cigarettes every day or some days are provided in eTable 4 and eTable 5 in the Supplement. The association of exposure to antismoking messages with smoking cessation intentions were largely preserved among those who smoked every day, but there was no association among those who smoked some days.

**Table 4.** Odds of Smoking Cessation Intentions and Attempts Among Current Smokers, by Scale of Exposure to Antismoking Advertisement Messages

Exposure to antismoking advertisements	Smoking cessation intention^a^		Smoking cessation attempted^b^	
	aOR (95% CI)^c^	*P* value	aOR (95% CI)^c^	*P* value
Exposure to messages				
Not seen	1 [Reference]	NA	1 [Reference]	NA
Single message type seen	1.22 (0.50-3.01)	.66	1.37 (0.64-2.92)	.42
Multiple message types seen	2.19 (1.10-4.34)	.03	1.31 (0.69-2.52)	.40
Cumulative exposure to messages				
Cumulative exposure to antismoking advertisement messages	1.21 (1.02-1.44)	.03	1.00 (0.84-1.20)	.98

Abbreviations: aOR, adjusted odds ratio; NA, not applicable.

^a^ Assessed using the survey question: “Are you seriously considering quitting smoking within the next 6 months?”

^b^ Assessed using the survey question: “At any time in the past year, have you stopped smoking for 1 day or longer because you were trying to quit?”

^c^ Adjusted for covariates including age, sex, household annual income, race/ethnicity, educational level, and rural or urban residence.

In calculating the numbers of smokers who would have quit intentions associated with exposure to multiple advertisements, we found that if all smokers were to be exposed to multiple antitobacco messages, there could be an estimated 3 980 880 current smokers (95% CI, 492 480-7 223 040) with a quit intention.

## Discussion

On full completion of the execution of the court-ordered tobacco industry antismoking advertising campaign on television and in newspapers and several months into the implementation of antismoking message placement on tobacco company corporate websites and cigarette package onserts, the reach of the corrective antismoking advertisements among US adults was 45.8% in the general adult population and 66.8% among current smokers. Although the coverage among smokers improved, population-level coverage represents only a small increment from a study that was conducted prior to the completion of the television advertisements and the onset of antismoking message placement on tobacco company websites and cigarette packages.^21^

This study found a significant association between exposure to corrective antismoking messages and intentions to quit cigarette smoking among a nationally representative sample of adult smokers in the United States. The findings build on previous research that found that exposure to antitobacco messaging is associated with an increased likelihood of smoking cessation intentions.^8,9,13^ However, prior research has focused largely on federally or state-sponsored and federally or state-executed antismoking advertising campaigns.^8,15,16,28^

Per the Transtheoretical Model (Stages of Change),^29,30,31^ behavioral intentions are the antecedent to behavioral change. As such, the observed association between exposure to antismoking advertisements and increased intentions to quit is an encouraging finding. Specifically, our study suggests that adult cigarette smokers who were exposed to antismoking advertisements were further along the continuum of behavior change toward cigarette smoking cessation (ie, contemplation and preparation) than those who were not exposed to these messages. This finding suggests that court-ordered industry-sponsored antismoking advertisements may be an effective component of comprehensive tobacco control measures^32^ by increasing intentions to quit among adult cigarette smokers. However, this campaign was not sufficient for behavior change, as evidenced by the lack of association with actual attempts to quit. Our findings are consistent with those of a 2012 study that showed that exposure to tobacco industry–sponsored campaigns was not associated with increased attempts to quit.^9^

The content of messages used in antismoking advertisements play a crucial role in stimulating behavioral outcomes.^15,18,28^ Our findings underscore the need to make corrective statements more emphatic than they currently are. The prosaic, unremarkable, and heavily scripted nature of the industry-sponsored antitobacco advertisements contrast quite sharply with their own pro-tobacco advertisements that are full of color, vibrancy, and designed to elicit strong emotional responses. Messages used in future industry-sponsored corrective advertising campaigns should undergo more rigorous pretesting, similar to other antitobacco advertisements, to ensure those messages resonate with the target audience.^33^ Furthermore, enhancing the present corrective statements with industry deception statements and firsthand emotional narrative testimonials from former smokers (where possible; eg, on tobacco company websites) may improve the effectiveness of industry-sponsored antismoking advertisement campaigns.^20^ These components have been shown to resonate highly among smokers, yield greater recall rates, and bring about changes in smoking behaviors.^15,19,20,33^

In addition, a comprehensive approach that combines this type of population-level strategy with individual-level interventions (eg, smoking cessation counseling by a health professional) may provide further benefits. Clinicians are advised to use effective counseling and medication in treating tobacco dependence, guided under approaches such as the 5As model.^34^ Population-level interventions can be potentiated such that individuals who have been exposed to corrective antismoking messages and are considering quitting (step 3 of the 5As model) can be referred to a nicotine dependence treatment program to receive assistance with quitting (step 4) and commence follow-up care (step 5).

Although interventions such as corrective statements, when combined with other robust tobacco control measures, have the potential to lower the overall prevalence of smoking, there is also potential for the interventions to have a negative equity outcome. In our study, we observed that exposure to these advertisements were significantly lower among ethnic/racial minority groups as well as those with a low educational level and low income. Equity approaches to public health interventions call for enhanced and sustained efforts to ensure that hard-to-reach populations receive culturally sensitive messages delivered in a medium and form that they can access, understand, and use.

### Limitations

This study has several limitations. First, HINTS data are cross-sectional; hence, causal inferences cannot be made. Second, HINTS data are self-reported, and several covariates, as well as the outcome variable used to assess attempts to quit, rely on respondents’ ability to recall past events; as such, this factor may have rendered our study prone to recall bias. Owing to the framing of the exposure question in the survey, we could not determine to which specific advertisement medium respondents were exposed (ie, television, newspapers, tobacco company corporate websites, or cigarette packages); as such, we could not assess exposure by individual advertising medium because these data were pooled. In addition, the primary outcomes of this study were examined only among current cigarette smokers, and thus we could not explore the outcome of antismoking advertisements among those who had recently quit smoking.

## Conclusions

The reach of court-ordered industry advertisements has increased among smokers; however, the reach of this advertisement campaign within the general population remains suboptimal, and disparities in its penetration persists among disadvantaged groups. Industry antismoking advertising campaigns may help improve intentions to quit smoking among adult cigarette smokers; however, they have not been effective in stimulating behavioral change among smokers. Strategies to improve the effectiveness of the industry-sponsored antismoking advertisement campaigns outlined in this study should be considered to achieve maximal tobacco control benefits.

## Notes

Supplement. **eTable 1.** Smoking Cessation Intentions and Attempts Among “Every Day” Smokers, by Sociodemographic Characteristics and Antismoking Advertisement Exposure **eTable 2.** Smoking Cessation Intentions and Attempts Among “Some Days” Smokers, by Sociodemographic Characteristics and Antismoking Advertisement Exposure **eTable 3.** Factors Associated with Exposure to US Federal Court-Ordered Antismoking Advertisements Based on Multivariable Logistic Regression **eTable 4.** Odds of Smoking Cessation Intentions and Attempts Among “Every Day” Smokers, by Scale of Exposure to Antismoking Advertisement Messages **eTable 5.** Odds of Smoking Cessation Intentions and Attempts Among “Some Days” Smokers, by Scale of Exposure to Antismoking Advertisement Messages

## References

[1] United States District Court for the District of Columbia. United States of America and interveners vs Philip Morris USA, Inc., et al. US district court for the District of Columbia. Civil action no. 99-2496 (GK). Updated 2018. Accessed December 12, 2018. https://www.tobaccofreekids.org/assets/content/what_we_do/industry_watch/doj/FinalOpinion.pdf

[2] Farber HJ, Neptune ER, Ewart GW Corrective statements from the tobacco industry: more evidence for why we need effective tobacco control. Ann Am Thorac Soc. 2018;15(2):127-130. doi:10.1513/AnnalsATS.201711-845GH 29140104

[3] Dyer O US tobacco companies run court ordered advertisements. BMJ. 2017;359:j5613. doi:10.1136/bmj.j5613 29196498

[4] Campaign for Tobacco-Free Kids Tobacco companies ordered to place statements about products’ dangers on websites and cigarette packs. Updated 2018. Accessed December 11, 2018. https://www.tobaccofreekids.org/press-releases/2018_05_01_correctivestatements

[5] World Health Organization *WHO Report on the Global Tobacco Epidemic, 2019: Offer Help to Quit Tobacco Use*. World Health Organization; 2019.

[6] World Health Organization WHO report on the global tobacco epidemic, 2017: monitoring tobacco use and prevention policies. Updated 2017. Accessed February 12, 2020. https://apps.who.int/iris/bitstream/handle/10665/255874/9789241512824-eng.pdf?sequence=1

[7] Siegel M Mass media antismoking campaigns: a powerful tool for health promotion. Ann Intern Med. 1998;129(2):128-132. doi:10.7326/0003-4819-129-2-199807150-00013 9669972

[8] Davis KC, Farrelly MC, Duke J, Kelly L, Willett J Antismoking media campaign and smoking cessation outcomes, New York state, 2003-2009. Prev Chronic Dis. 2012;9:E40. 22261250 PMC3320091

[9] Emery S, Kim Y, Choi YK, Szczypka G, Wakefield M, Chaloupka FJ The effects of smoking-related television advertising on smoking and intentions to quit among adults in the United States: 1999-2007. Am J Public Health. 2012;102(4):751-757. doi:10.2105/AJPH.2011.300443 22397350 PMC3489369

[10] Li L, Borland R, Yong HH, Reported exposures to anti-smoking messages and their impact on Chinese smoker’s subsequent quit attempts. Int J Behav Med. 2014;21(4):667-676. doi:10.1007/s12529-013-9349-3 24078490 PMC4546845

[11] Li L, Borland R, Yong HH, The association between exposure to point-of-sale anti-smoking warnings and smokers’ interest in quitting and quit attempts: findings from the International Tobacco Control Four Country Survey. Addiction. 2012;107(2):425-433. doi:10.1111/j.1360-0443.2011.03668.x 21954921 PMC3260376

[12] Liu H, Tan W The effect of anti-smoking media campaign on smoking behavior: the California experience. Ann Econ Financ. 2009;10(1):29-47. Accessed May 21, 2020. http://www.aeconf.com/Articles/May2009/aef100103.pdf

[13] Centers for Disease Control and Prevention (CDC) Antismoking messages and intention to quit—17 countries, 2008-2011. MMWR Morb Mortal Wkly Rep. 2013;62(21):417-422. 23718949 PMC4604857

[14] Henriksen L, Dauphinee AL, Wang Y, Fortmann SP Industry sponsored anti-smoking ads and adolescent reactance: test of a boomerang effect. Tob Control. 2006;15(1):13-18. doi:10.1136/tc.2003.006361 16436398 PMC2563637

[15] Leas EC, Myers MG, Strong DR, Hofstetter CR, Al-Delaimy WK Recall of anti-tobacco advertisements and effects on quitting behavior: results from the California Smokers Cohort. Am J Public Health. 2015;105(2):e90-e97. doi:10.2105/AJPH.2014.302249 25521871 PMC4318324

[16] Biener L, McCallum-Keeler G, Nyman AL Adults’ response to Massachusetts anti-tobacco television advertisements: impact of viewer and advertisement characteristics. Tob Control. 2000;9(4):401-407. doi:10.1136/tc.9.4.401 11106710 PMC1748390

[17] Wakefield MA, Spittal MJ, Yong HH, Durkin SJ, Borland R Effects of mass media campaign exposure intensity and durability on quit attempts in a population-based cohort study. Health Educ Res. 2011;26(6):988-997. doi:10.1093/her/cyr054 21730252 PMC3219882

[18] Biener L, Ji M, Gilpin EA, Albers AB The impact of emotional tone, message, and broadcast parameters in youth anti-smoking advertisements. J Health Commun. 2004;9(3):259-274. doi:10.1080/10810730490447084 15360037

[19] McAfee T, Davis KC, Shafer P, Patel D, Alexander R, Bunnell R Increasing the dose of television advertising in a national antismoking media campaign: results from a randomised field trial. Tob Control. 2017;26(1):19-28. doi:10.1136/tobaccocontrol-2015-052517 26678518 PMC5108680

[20] Lee SJ, Sanders-Jackson A, Tan ASL Effects of current and enhanced tobacco corrective messages on smokers’ intention to quit smoking and intention to purchase cigarettes. Nicotine Tob Res. 2020;22(4):569-575. doi:10.1093/ntr/ntz063 31045214 PMC13396259

[21] Chido-Amajuoyi OG, Yu RK, Agaku I, Shete S Exposure to court-ordered tobacco industry antismoking advertisements among US adults. JAMA Netw Open. 2019;2(7):e196935-e196935. doi:10.1001/jamanetworkopen.2019.6935 31298713 PMC6628589

[22] National Cancer Institute Health Information National Trends Survey 5 (HINTS 5) cycle 2 methodology report. Published July 2018. Accessed March/8, 2019. https://hints.cancer.gov/docs/methodologyreports/HINTS5_Cycle_2_Methodology_Report.pdf

[23] von Elm E, Altman DG, Egger M, Pocock SJ, Gøtzsche PC, Vandenbroucke JP; STROBE Initiative The Strengthening the Reporting of Observational Studies in Epidemiology (STROBE) statement: guidelines for reporting observational studies. Ann Intern Med. 2007;147(8):573-577. doi:10.7326/0003-4819-147-8-200710160-00010 17938396

[24] American Association for Public Opinion Research Standard definitions: final dispositions of case codes and outcome rates for surveys. Updated 2016. Accessed April 30, 2019. https://www.aapor.org/AAPOR_Main/media/publications/Standard-Definitions20169theditionfinal.pdf

[25] Finney Rutten LJ, Blake KD, Skolnick VG, Davis T, Moser RP, Hesse BW Data resource profile: the National Cancer Institute’s Health Information National Trends Survey (HINTS). Int J Epidemiol. 2020;49(1):17-17j. doi:10.1093/ije/dyz083 31038687 PMC7124481

[26] National Cancer Institute Health Information National Trends Survey 5 (HINTS 5): cycle 3 methodology report. Published August 2019 Accessed May 22, 2020. https://hints.cancer.gov/docs/Instruments/HINTS5_Cycle3_MethodologyReport.pdf

[27] Creamer MR, Wang TW, Babb S, Tobacco product use and cessation indicators among adults—United States, 2018. MMWR Morb Mortal Wkly Rep. 2019;68(45):1013-1019. doi:10.15585/mmwr.mm6845a2 31725711 PMC6855510

[28] McAfee T, Davis KC, Alexander RL Jr, Pechacek TF, Bunnell R Effect of the first federally funded US antismoking national media campaign. Lancet. 2013;382(9909):2003-2011. doi:10.1016/S0140-6736(13)61686-4 24029166

[29] Prochaska JO, Velicer WF The transtheoretical model of health behavior change. Am J Health Promot. 1997;12(1):38-48. doi:10.4278/0890-1171-12.1.38 10170434

[30] Littell JH, Girvin H Stages of change: a critique. Behav Modif. 2002;26(2):223-273. doi:10.1177/0145445502026002006 11961914

[31] Prochaska JO, Redding CA, Evers KE The transtheoretical model and stages of change In: Glanz K, Rimer BK, Viswanath KV, eds. Health Behavior: Theory, Research, and Practice. Jossey-Bass; 2015:125-148.

[32] Centers for Disease Control and Prevention Best practices for comprehensive tobacco control programs—2014. Accessed April 8, 2020. https://www.cdc.gov/tobacco/stateandcommunity/best_practices/index.htm

[33] Centers for Disease Control and Prevention Best practices user guides: health communications in tobacco prevention and control. Updated 2018. Accessed April 8, 2020. https://www.cdc.gov/tobacco/stateandcommunity/bp-health-communications/pdfs/health-communications-508.pdf

[34] Fiore MC, Bailey WC, Cohen SJ, Treating Tobacco Use and Dependence: Clinical Practice Guideline. US Department of Health and Human Services; 2000.

