# Supplementary material for: Association of Exposure to Court-Ordered Tobacco Industry Antismoking Advertisements With Intentions and Attempts to Quit Smoking Among US Adults
Source: JAMA Netw Open. 2020 Jul 7;3(7):e209504. doi: 10.1001/jamanetworkopen.2020.9504 (PMC7341176; doi:10.1001/jamanetworkopen.2020.9504)
Supplement: Supplement. — eTable 1. Smoking Cessation Intentions and Attempts Among “Every Day” Smokers, by Sociodemographic Characteristics and Antismoking Advertisement Exposure eTable 2. Smoking Cessation Intentions and Attempts Among “Some Days” Smokers, by Sociodemographic Characteristics and Antismoking Advertisement Exposure eTable 3. Factors Associated with Exposure to US Federal Court-Ordered Antismoking Advertisements Based on Multivariable Logistic Regression eTable 4. Odds of Smoking Cessation Intentions and Attempts Among “Every Day” Smokers, by Scale of Exposure to Antismoking Advertisement Messages eTable 5. Odds of Smoking Cessation Intentions and Attempts Among “Some Days” Smokers, by Scale of Exposure to Antismoking Advertisement Messages [file jamanetwopen-3-e209504-s001.pdf]

Supplementary Online Content

Chido-Amajuoyi OG, Agaku I, Mantey DS, Yu RK, Shete S. Association of exposure to court-ordered tobacco industry antismoking advertisements with intentions and attempts to quit smoking among US adults. *JAMA Netw Open.* 2020;3(7):e209504. doi:10.1001/jamanetworkopen.2020.9504

eTable 1. Smoking Cessation Intentions and Attempts Among "Every Day" Smokers, by Sociodemographic Characteristics and Antismoking Advertisement Exposure
eTable 2. Smoking Cessation Intentions and Attempts Among "Some Days" Smokers, by Sociodemographic Characteristics and Antismoking Advertisement Exposure
eTable 3. Factors Associated with Exposure to US Federal Court-Ordered Antismoking Advertisements Based on Multivariable Logistic Regression
eTable 4. Odds of Smoking Cessation Intentions and Attempts Among “Every Day” Smokers, by Scale of Exposure to Antismoking Advertisement Messages
eTable 5. Odds of Smoking Cessation Intentions and Attempts Among “Some Days” Smokers, by Scale of Exposure to Antismoking Advertisement Messages

This supplementary material has been provided by the authors to give readers additional information about their work.

© 2020 Chido-Amajuoyi OG et al. *JAMA Network Open.*

eTable 1. Smoking Cessation Intentions and Attempts Among "Every Day" Smokers, by Sociodemographic Characteristics and Antismoking Advertisement Exposure

Characteristics	Smoking cessation intention					Smoking cessation attempted			
	Overall Sample	Yes[a]				Overall Sample	Yes[b]		
	n	%	95% CI			*n*	%	95% CI	
**Total**	421	57.5	50.0	65.1		426	53.9	47.5	60.2
**Sex**									
Male	187	58.6	47.1	70.1		189	58.4	48.0	68.7
Female	234	56.4	45.7	67.0		237	48.9	38.7	59.1
**Age, years**									
18 to 34	25	40.2	16.2	64.2		25	59.6	35.8	83.3
35 to 49	78	69.1	55.4	82.9		79	54.7	39.4	70.1
50 to 64	204	56.3	44.7	67.9		205	54.6	46.0	63.3
65+	114	48.1	33.9	62.3		116	46.5	32.0	61.1
**Race/ethnicity**									
Non-Hispanic White	251	56.7	47.0	66.3		252	51.4	41.9	61.0
Non-Hispanic Black	57	54.4	36.5	72.3		57	60.9	42.9	78.9
Hispanic	44	48.8	26.7	70.9		44	56.3	30.3	82.3
**Level of education**									
High school graduate or lower	171	58.3	47.6	69.1		174	46.2	36.7	55.7
Post high school/some college	156	55.3	42.5	68.2		157	60.9	48.6	73.1

© 2020 Chido-Amajuoyi OG et al. *JAMA Network Open.*

College graduate/postgraduate	92	62.7	45.7	79.7		93	58.8	41.3	76.3
**Residence**									
Urban	346	59.5	51.6	67.3		350	57.7	51.5	63.9
Rural	75	49.2	30.7	67.7		76	37.5	21.7	53.2
**Household Annual income**									
Less than $35,000	211	57.0	43.8	70.1		212	57.3	46.9	67.6
$35,000 to $49,999	60	54.4	33.1	75.7		60	57.6	37.2	78.0
$50,000 to $74,999	52	56.3	34.0	78.5		53	42.6	23.8	61.5
$75,000 or more	68	64.9	49.4	80.4		69	55.9	39.6	72.1
**Exposure to antismoking advertisements**									
Not Seen	128	46.8	34.8	58.7		130	45.9	33.6	58.1
Single message type seen	83	52.9	36.5	69.2		84	53.5	38.1	68.9
Multiple message types seen	194	65.1	55.1	75.1		195	57.0	46.6	67.5
**Exposure to antismoking advertisements**									
Number of Message Seen (mean; 95%CI)	405	1.9	1.7	2.2		409	1.7	1.5	2.0

[a] Indicates "yes" response to the survey question: “Are you seriously considering quitting smoking within the next six months?”

[b] Indicates "yes" response to the survey question: “At any time in the past year, have you stopped smoking for one day or longer because you were trying to quit?”

© 2020 Chido-Amajuoyi OG et al. *JAMA Network Open.*

eTable 2. Smoking Cessation Intentions and Attempts Among "Some Days" Smokers, by Sociodemographic Characteristics and Antismoking Advertisement Exposure

**Characteristics**									
	**Smoking cessation intention**					**Smoking cessation attempted**			
	**Overall Sample**	**Yes**[a]				**Overall Sample**	**Yes**[b]		
	n	%	95% CI			***n***	%	95% CI	
**Total**	178	65.7	53.2	78.1		180	75.9	64.5	87.4
**Sex**									
Male	93	62.8	44.4	81.1		93	69.3	51.4	87.2
Female	85	69.9	52.3	87.6		87	85.5	74.7	96.4
**Age, years**									
18 to 34	32	58.9	31.2	86.6		32	84.8	69.1	100.0
35 to 49	44	68.5	42.0	95.1		44	72.0	44.3	99.7
50 to 64	61	65.1	45.5	84.8		61	71.4	52.3	90.5
65+	36	88.5	76.3	100.0		37	90.9	78.9	100.0
**Race/ethnicity**									
Non-Hispanic White	85	51.1	35.0	67.2		85	64.2	46.4	82.0
Non-Hispanic Black	32	67.4	36.4	98.4		32	78.2	50.8	100.0
Hispanic	27	87.8	75.2	100.0		28	87.1	73.5	100.0
**Level of education**									
High school graduate or lower	42	60.6	34.7	86.4		43	65.8	39.8	91.8
Post high school/some college	65	68.5	52.1	84.9		66	76.0	59.8	92.1

© 2020 Chido-Amajuoyi OG et al. *JAMA Network Open.*

College graduate/postgraduate	68	62.6	37.6	87.6		68	82.0	67.9	96.0
**Residence**									
Urban	160	67.7	55.0	80.5		162	80.4	70.9	90.0
Rural	18	55.1	0.0	100.0		18	53.0	0.0	100.0
**Household Annual income**									
Less than $35,000	70	58.0	34.3	81.7		70	71.4	47.8	95.1
$35,000 to $49,999	26	94.6	87.4	100.0		27	94.7	87.5	100.0
$50,000 to $74,999	16	87.9	69.0	100.0		16	91.8	80.0	100.0
$75,000 or more	49	59.4	33.6	85.1		49	70.1	47.4	92.7
**Exposure to antismoking advertisements**									
Not Seen	67	63.4	41.2	85.6		68	74.8	51.7	97.8
Single message type seen	37	61.0	29.5	92.5		37	81.3	61.7	100.0
Multiple message types seen	69	67.9	52.9	82.8		70	70.3	51.3	89.3
**Exposure to antismoking advertisements**									
Number of Message Seen (mean; 95%CI)	173	1.4	1.0	1.8		175	1.3	0.9	1.7

[a] Indicates "yes" response to the survey question: “Are you seriously considering quitting smoking within the next six months?”

[b] Indicates "yes" response to the survey question: “At any time in the past year, have you stopped smoking for one day or longer because you were trying to quit?”

© 2020 Chido-Amajuoyi OG et al. *JAMA Network Open.*

eTable 3. Factors Associated with Exposure to US Federal Court-Ordered Antismoking Advertisements Based on Multivariable Logistic Regression

**Characteristics**	aOR[a]	**95% CI**		*p*
**Sex**				
Male	Ref			
Female	0.95	0.76	1.19	0.6290
**Age, y**				
18-34	Ref			
35-49	0.57	0.40	0.82	0.0027
50-64	0.79	0.55	1.15	0.2200
≥65	0.79	0.54	1.14	0.2020
**Race/ethnicity**				
Non-Hispanic white	Ref			
Non-Hispanic black	0.99	0.72	1.35	0.9360
Hispanic	0.98	0.72	1.33	0.9040
**Level of education**				
College graduate or postgraduate	Ref			NA
Post-high school or some college	1.11	0.91	1.36	0.2940
≤High school graduate	0.91	0.67	1.23	0.5170
**Residence**				
Urban	Ref			
Rural	1.05	0.77	1.42	0.7620
**Household annual income, $**				
<35,000	Ref			
35,000-49,999	1.36	0.91	2.02	0.1340

© 2020 Chido-Amajuoyi OG et al. *JAMA Network Open.*

50,000-74,999	1.31	0.92	1.87	0.1370
≥75,000	1.81	1.33	2.48	0.0004
**Smoking status**				
Never	Ref			
Former	1.39	1.08	1.79	0.0106
Current	3.55	2.60	4.83	<0.0001
[a] Adjusted odds ratio; adjusted for covariates including age, sex, household annual income, race /ethnicity, level of education and rural-urban residence.				

© 2020 Chido-Amajuoyi OG et al. *JAMA Network Open.*

eTable 4. Odds of Smoking Cessation Intentions and Attempts Among "Every Day" Smokers, by Scale of Exposure to Antismoking Advertisement Messages

**Exposure to antismoking advertisements**	**Smoking cessation intention[a]**					**Smoking cessation attempted[b]**			
	aOR[c]	95% CI		*p*		aOR[c]	95% CI		*p*
**Panel A**									
Not Seen	1.00					1.00			
Single message type seen	1.53	0.62	3.77	0.3447		1.58	0.62	4.01	0.3305
Multiple message types seen	2.90	1.42	5.92	0.0043		1.75	0.75	4.09	0.1898
**Panel B**									
Cumulative exposure to antismoking advertisement messages	1.24	1.05	1.47	0.0137		1.01	0.81	1.25	0.9516

[a] Assessed using the survey question: "Are you seriously considering quitting smoking within the next six months?"

[b] Assessed using the survey question: "At any time in the past year, have you stopped smoking for one day or longer because you were trying to quit?"

[c] Adjusted odds ratio; adjusted for covariates including age, sex, household annual income, race /ethnicity, level of education and rural-urban residence.

© 2020 Chido-Amajuoyi OG et al. *JAMA Network Open.*

eTable 5. Odds of Smoking Cessation Intentions and Attempts Among "Some Days" Smokers, by Scale of Exposure to Antismoking Advertisement Messages

**Exposure to antismoking advertisements**	**Smoking cessation intention[a]**					**Smoking cessation attempted[b]**			
	aOR[c]	95% CI		*p*		aOR[c]	95% CI		*p*
**Panel A**									
Not Seen	1.00					1.00			
Single message type seen	0.64	0.09	4.50	0.6450		0.51	0.04	6.65	0.6001
Multiple message types seen	1.16	0.25	5.45	0.8450		0.36	0.06	2.02	0.2390
**Panel B**									
Cumulative exposure to antismoking advertisement messages	1.21	0.78	1.90	0.3904		0.91	0.61	1.36	0.6289

[a] Assessed using the survey question: "Are you seriously considering quitting smoking within the next six months?"

[b] Assessed using the survey question: "At any time in the past year, have you stopped smoking for one day or longer because you were trying to quit?"

[c] Adjusted odds ratio; adjusted for covariates including age, sex, household annual income, race /ethnicity, level of education and rural-urban residence.

© 2020 Chido-Amajuoyi OG et al. *JAMA Network Open.*
